# Left atrial conduction times and regional velocities in persistent atrial fibrillation patients with and without fibrotic atrial cardiomyopathy

**DOI:** 10.1007/s00380-023-02282-5

**Published:** 2023-07-07

**Authors:** Emanuel Heil, Jin-Hong Gerds-Li, Julian Keznickl-Pulst, Vesna Furundzija-Cabraja, Felix Hohendanner, Leif-Hendrik Boldt, Philipp Stawowy, Doreen Schoeppenthau

**Affiliations:** 1 Deutsches Herzzentrum der Charité, Charité Universitätsmedizin Berlin, Augustenburger Platz 1, 13353 Berlin, Germany; 2 Deutsches Zentrum für Herz-Kreislauf-Forschung, Standort Berlin, Berlin, Germany

**Keywords:** Atrial fibrillation, Fibrotic atrial cardiomyopathy, Atrial substrate, Left atrial conduction, Voltage mapping, Catheter ablation

## Abstract

Despite the progress in understanding left atrial substrate and arrhythmogenesis, only little is known about conduction characteristics in atrial fibrillation patients with various stages of fibrotic atrial cardiomyopathy (FACM). This study evaluates left atrial conduction times and conduction velocities based on high-density voltage and activation maps in sinus rhythm (CARTO^®^3 V7) of 53 patients with persistent atrial fibrillation (LVEF 60% (55–60 IQR), LAVI 39 ml/m^2^ (31–47 IQR), LApa 24 ± 6 cm^2^). Measurements were made in low voltage areas (LVA ≤ 0.5 mV) and normal voltage areas (NVA ≥ 1.5 mV) at the left atrial anterior and posterior walls. Maps of 28 FACM and 25 no FACM patients were analyzed (19 FACM I/II, 9 FACM III/IV, LVA 14 ± 11 cm^2^). Left atrial conduction time averaged to 110 ± 24 ms but was shown to be prolonged in FACM (119 ms, + 17%) when compared to no FACM patients (101 ms, *p* = 0.005). This finding was pronounced in high-grade FACM (III/IV) (133 ms, + 31.2%, *p* = 0.001). In addition, the LVA extension correlated significantly with the left atrial conduction time (*r* = 0.56, *p* = 0.002). Conduction velocities were overall slower in LVA than in NVA (0.6 ± 0.3 vs. 1.3 ± 0.5 m/s, -51%, p < 0.001). Anterior conduction appeared slower than posterior, which was significant in NVA (1 vs. 1.4 m/s, -29%, *p* < 0.001) but not in LVA (0.6 vs. 0.8 m/s, *p* = 0.096). FACM has a significant influence on left atrial conduction characteristics in patients with persistent atrial fibrillation. Left atrial conduction time prolongs with the grade of FACM and the quantitative expanse of LVA up to 31%. LVAs show a 51% conduction velocity reduction compared to NVA. Moreover, regional conduction velocity differences are present in the left atrium when comparing anterior to posterior walls. Our data may influence individualized ablation strategies.

## Introduction

Atrial fibrillation (AF) is the most common human arrhythmia and is associated with an increased risk for cardiovascular mortality and morbidity. Since the pulmonary veins (PV) are important trigger sites of paroxysmal AF [1], pulmonary vein isolation (PVI) is a highly effective treatment. However, in more advanced stages, such as persistent AF, catheter ablation is challenging since arrhythmogenic substrates and mechanisms besides PV triggers, including atrial fibrosis, seem to gain a critical role [2–4]. Slow conduction and functional blocks impact reentry formation, propagation, and in this way, AF maintenance [5]. Oakes et al. reported that AF recurrence after PV antrum isolation is likely to occur in patients with low-voltage zones [3]. Fibrotic atrial cardiomyopathy (FACM) is more frequent in persistent AF [2, 6, 7], and it is well-known that PVI alone leads to suboptimal clinical outcomes in those patients [8–10]. Intraprocedural characterization of the atrial substrate was performed by mapping the peak-to-peak amplitude of bipolar electrograms, and a voltage lower than 0.5 mV was shown to identify fibrosis [11–13]. New ablation strategies targeting low voltage areas (LVA) [4, 6, 14–18] were introduced in the last years to improve efficacy but have yet to be proven. However, a first randomized trial in persistent AF patients (ERASE-AF) has recently shown improved outcomes with combined PVI and individualized ablation of low voltage areas [19].

Little is known about the conduction velocities (CV) of low and normal voltage areas in sinus rhythm. However, Sato et al. indicated that reduced atrial CV might be associated with AF recurrence after catheter ablation [20]. Therefore, the present study was conducted to examine how different grades of fibrosis and bipolar electrogram amplitudes (voltage < 0.5 mV, voltage > 1.5 mV) impact total left atrial conduction times and affect local conduction velocities in the anterior and posterior left atrium (LA). A deeper understanding of LA conduction might influence future individualized ablation concepts to further improve outcomes in those patients.

## Materials and methods

### Study population

This retrospective single-center observational study analyzed 53 high-density maps of persistent AF patients (clinical definition) undergoing radiofrequency catheter ablation using the CARTO3^®^ V7 navigation system (Biosense Webster, Diamond Bar, CA) from 2019 to 2021. Only maps acquired in sinus rhythm with more than 1000 reading points and well-distributed anatomical coverage were included. Exclusively de novo ablations and cases without previous substrate modification were accepted. All patients provided informed consent for the electrophysiologic study and ablation. Patients are part of the ABAF study population (ethics committee votum EA2/249/18).

### Electrophysiological study and ablation

#### Electroanatomic mapping

At the beginning of the procedure, electric cardioversion was performed to restore sinus rhythm if needed. To conduct a simultaneous high-density activation and voltage mapping, a 10-polar circular mapping catheter (Lasso Nav, Biosense Webster Inc, Diamond Bar, CA) with a cardiac tissue proximity filter based on catheter location and impedance measurements was used. A cycle length (5%) and position stability filter (4 mm) were utilized to avoid extrasystole beat detections. The maximum negative deflection (– dV/dt) in each distal unipolar signal was used for the local activation time (LAT) calculation.

#### Left atrial substrate and conduction properties measurements

**Fig. 1:**
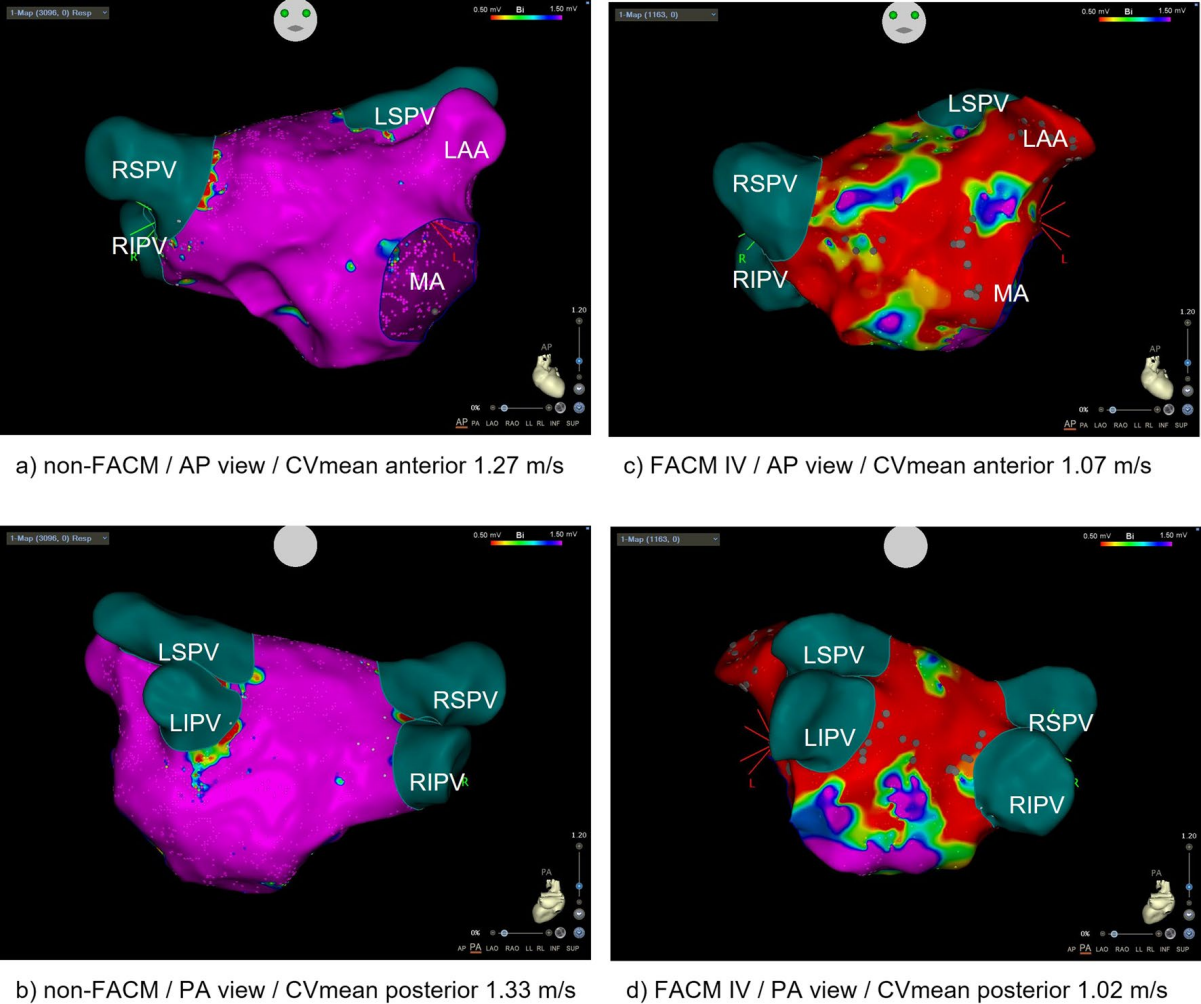
CARTO-reconstruction of left atrial voltage map in non-FACM (**a/b**) and FACM IV (**c/d**) in AP (**a/c**) and PA (**b/d**) view. *FACM* Fibrotic atrial cardiomyopathy, *CV* conduction velocity, *AP* anterior–posterior, *PA* posterior–anterior, *RSPV* right superior pulmonary vein, *RIPV* right inferior pulmonary vein, *LSPV* left superior pulmonary vein, *LIPV* left inferior pulmonary vein, *LAA* left atrial appendage

We defined voltage thresholds < 0.5 mV as severe fibrosis (low voltage area, LVA), sites with voltages of 0.5–1.5 as mild/moderate fibrosis, and areas with > 1.5 mV as non-fibrotic myocardium (normal voltage area, NVA). Scar was delineated as a zone without a discrete bipolar electrogram apart from far-field or noise. LVA was registered if ≥ 3 mapping points with voltage < 0.5 mV were adjacent within a mapping distance < 1 cm^2^. The LVA size was measured with the CARTO area measurement function excluding the pulmonary vein regions and the mitral annulus. The FACM was rated by the classification after Kottkamp et al. [6, 16, 21–23]. In detail, this means the following: no FACM: no detectable voltage area < 1.5 mV. FACM I: very limited severe fibrosis but detection of adjacent confluent moderately reduced voltage areas (0.5–1.5 mV) with locally fragmented electrograms. FACM II: 1(-2) confluent LVAs, FACM III: pronounced left atrial fibrosis but still regionally confined with ≥ 2 large LVAs, FACM IV (“strawberry”): diffuse fibrosis with large LVAs leaving some small moderately diseased regions, hardly any voltage > 1.5 mV “ [16]. Evading observer bias, the FACM grading was reviewed by a second physician blinded to clinical and periinterventional data. FACM I and II were defined as low-grade, and FACM III and IV as high-grade disease (cf. Fig. 1).

Total left atrial conduction time (LACT) was defined as the difference between the latest LAT (mostly lateral mitral annulus) minus the earliest LAT (mainly Bachmann bundle region). Local activation times were taken as the average from triads of sites. Local CVs were measured in the left atrial anterior and posterior walls and cataloged according to the area’s voltage thresholds (LVA or NVA). For this purpose, CV was defined as the linear distance between each pair of points divided by their difference in activation times and was calculated explicitly along the activation front using the coherent algorithm. Multiple measurements were averaged to avoid confounders.

#### Catheter ablation

Pulmonary vein antrum isolation was performed with a 3.5 mm open‐irrigated contact force ablation catheter (Smart Touch; Biosense Webster Inc, CA). Ablation was guided by the ablation index (AI target 550 anterior, 450 posterior) using a power limit of 35 W (reduction to 25 W at the LA posterior wall). Automatic ablation point acquisition settings were used (VisiTag module, Biosense Webster, CA, USA). The primary endpoint of the ablation procedure was electrical PV isolation with the elimination or dissociation of PV potentials. Additional substrate modification based on voltage mapping was at the operator's discretion. Patients were followed up by the clinic or the referring doctor regularly at the 3-, 6- and 12-month mark after ablation with 24 h-Holter-ECGs or earlier in case of symptoms.

### Statistical methods

In statistical evaluation, metrical data are reported as mean ± standard deviation, ordinal and non-normally distributed items as median with interquartile range (IQR), and categorical variables in percentage. Homoscedasticity was evaluated by Leven- and *F* tests. Variation amongst interval-scaled subgroups was assessed and valued by *t*, Mann–Whitney *U*, and Kruskal–Wallis tests. Chi-Square- and Fisher’s Exact tests were used in nominal or ordinal scaled items to detect deviation. Furthermore, we conducted a one-way ANOVA to assess the effects of FACM on LACT. Eta and epsilon square were used to measure effect size [24]. Linear correlation was assessed by Pearson and Spearman correlation when appropriate. The guidelines of Cohen [25] were used to estimate the extent and power of a correlation. As recommended, *r* = 0.10, *r* = 0.30, and *r* = 0.50 were considered small, medium, and large in the correlation magnitude. A *p* value of ≤ 0.05 was considered statistically significant. All analyses were conducted with SPSS Statistic for Windows (version 29.0; SSS Inc, Chicago, IL) and Microsoft 365 Excel Apps for Enterprise (version 2206; Microsoft, Redmond, WA).

## Results

### General patient characteristics

Our study population (*n* = 53) consists of 31 males (58.5%) and 22 females (41.5%) with an average age of 66.8 ± 10.1 years and a mean body mass index (BMI) of 27.9 ± 4.2. Median CHA_2_DS_2_-VASc score was 3 (1.5–3.5 IQR). In their history, five patients (9.4%) reported a prior stroke. Regarding cardiovascular risk factors, 67.9% of the population presented with hypertension and 15% with diabetes mellitus. Coronary artery disease was diagnosed in 26.4% of cases. Left atrial and left ventricular dimensions were assessed by transthoracic echocardiography. The left atrium volume index (LAVI) averaged 38.76 ml/m^2^ (31–47 IQR), and the left atrial planimetric area (LA) to 24.1 ± 6.0 cm^2^. The median left ventricular ejection fraction (LVEF) was measured at 60% (55–60 IQR). The median NTproBNP level was determined in blood work with 634 pg/ml (282–1589 IQR). The mean glomerulation filtration rate (GFR) was estimated at 77.7 ± 19.2 ml/min/1.73 m^2^. Detailed patient characteristics are presented in Table 1.

**Table 1:** Summary of the physical and clinical characteristics of all included patients

Clinical parameter (*n* = 53)	
Age (years)	66.83 ± 10.11
Female, n (%)	22 (42)
BMI (kg/m^2^)	27.93 ± 4.20
CHA_2_DS_2_–VASc score	3 (1.5–3.5)
Hypertension, n (%)	36 (68)
Diabetes mellitus type II, n (%)	8 (15)
Coronary heart disease, n (%)	14 (26)
LVEF (%)	60 (55–60)
LAVI (ml/m^2^)	38.76 (31–47)
LA (cm^2^)	24.14 ± 6.04
GFR (ml/min/1.73m^2^)	77.7 ± 19.24
NTproBNP (pg/ml)	634 (282–1589)

*LVEF* left ventricular ejection fraction, *LAVI* left atrial volume index, *LA* Left atrial planimetric area, *GFR* glomerular filtration rate, *NTproBNP* N-terminal prohormone of brain natriuretic peptide

### Grading of FACM and clinical characteristics of FACM vs. no FACM patients

High-density electroanatomical voltage and activation mapping was performed with a median of 1411 points (1260.5–2341.5 IQR, min 1014–max 5806). Based on the sinus rhythm voltage maps, FACM was diagnosed in 28 patients (53%). Of these, 19 presented low-grade FACM (68%) and nine high-grade diseases (32%). The differences in clinical characteristics between no FACM and FACM patients are presented in Table 2.

**Table 2:** Patient characteristics in different FACM classes

	FACM 0	FACMI I–II	FACMI III–IV	FACM I–IV	0 vs. I–IV	0 vs. I–II	0 vs. III–IV	I–II vs. III–IV
	*n* = 25	*n* = 19	*n* = 9	*n* = 28	*p* value			
Clinical patient parameters								
Age (years)	63.04 ± 9.45	69.68 ± 10.76	71.33 ± 7.02	70.21 ± 9.61	0.004	0.020	0.011	0.340
Female, n (%)	4 (16)	12 (63)	6 (67)	18 (64)	< 0.001	0.002	0.009	0.878
BMI (kg/m^2^)	28.74 ± 4.35	27.63 ± 3.34	26.33 ± 5.25	27.21 ± 4.00	0.094	0.180	0.093	0.216
CHA_2_DS_2_–VASc score	2 (0.5–3)	3 (2–4)	3 (2.5–4.5)	3 (2–4)	0.314	0.193	0.293	0.151
Hypertension, n (%)	15 (60)	14 (74)	7 (78)	21 (75)	0.243	0.343	0.339	0.815
Diabetes mellitus type II, n (%)	3 (12)	3 (16)	2 (22)	5 (18)	0.584	0.735	0.502	0.699
Coronary heart disease, n (%)	6 (24)	6 (32)	2 (22)	8 (29)	0.706	0.576	0.914	0.609
LVEF (%)	60 (52–60)	60 (55–60)	60 (57.5–61)	60 (55–60)	0.233	0.377	0.141	0.211
LAVI (ml/m^2^)	36.5 (28–41.4)	45 (35–64)	41.06 (30–47)	42.34 (33.9–56.9)	0.008	0.009	0.182	0.050
LA (cm^2^)	23.50 ± 4.74	24.81 ± 7.02	24.50 ± 7.94	24.73 ± 7.09	0.259	0.254	0.351	0.465
GFR (ml/min/1.73m^2^)	83.54 ± 18.26	71.21 ± 22.33	75.19 ± 8.39	72.49 ± 18.89	0.018	0.025	0.040	0.306
NTproBNP (pg/ml)	297 (161–1177)	1433 (324–1989)	634 (336–1587.5)	1402.5 (330.5–1738)	0.014	0.017	0.175	0.251
Electrophysiological parameters								
LVA size (cm^2^)	NA	8.92 ± 7.80	23.64 ± 8.86	13.65 ± 10.63	NA	NA	NA	< 0.001
LACT (ms)	101.40 ± 20.70	113.32 ± 22.91	133.14 ± 26.69	118.65 ± 25.08	0.005	0.039	0.001	0.036
CV LVA (m/s)	NA	0.47 ± 0.24	0.77 ± 0.33	0.65 ± 0.33	NA	NA	NA	0.090
CV NVA (m/s)	1.25 ± 0.38	1.53 ± 0.72	1.27 ± 0.84	1.48 ± 0.74	0.117	0.011	0.085	0.201
CV anterior (m/s)	1.11 ± 0.34	0.98 ± 0.62	0.71 ± 0.36	0.89 ± 0.56	0.049	0.182	0.002	0.110
CV posterior (m/s)	1.38 ± 0.37	1.62 ± 0.82	1.14 ± 0.68	1.44 ± 0.80	0.372	0.103	0.090	0.060
CV NVA anterior (m/s)	1.11 ± 0.34	1.40 ± 0.52	0.54 ± 0.14	1.28 ± 0.57	0.164	0.024	0.014	0.022
CV NVA posterior (m/s)	1.38 ± 0.37	1.62 ± 0.82	1.56 ± 0.84	1.61 ± 0.81	0.116	0.132	0.334	0.440
CV LVA anterior (m/s)	NA	0.47 ± 0.24	0.75 ± 0.39	0.60 ± 0.34	NA	NA	NA	0.039
CV LVA posterior (m/s)	NA	NA	0.80 ± 0.26	0.80 ± 0.26	NA	NA	NA	NA

*FACM* fibrotic atrial cardiomyopathy, *BMI* body mass index, *LVEF* left ventricular ejection fraction, *LAVI* left atrial volume index, *LA* left atrial planimetric area, *GFR* glomerular filtration rate; *NTproBNP* N-terminal prohormone of brain natriuretic peptide, *LVA* low voltage area, *LACT* left atrial conduction time, *CV* conduction velocities, *NA* not available

Patients with detection of FACM were significantly older (63.0 ± 9.5 vs. 70.2 ± 9.6 years; *p* = 0.004) and more likely to be female (16.0% vs. 64.3%; *p* < 0.001; *r* = 0.62). According to the latter result, 21 males (67.7% of all males) but only four females (18.2% of all females) presented no FACM. In contrast, FACM I was diagnosed in four males (12.9%) and four females (18.2%), while FACM II was present in three males (9.7%) and eight women (38.1%). Similarly, high-grade disease was predominantly diagnosed in women since three males (9.7%) and three females (14.3%) met the criteria for FACM III, and three females (14.3%), while no males were diagnosed with FACM IV. Moreover, in FACM patients, the NTproBNP levels (297 vs. 634 pg/ml, *p* = 0.014) and the LAVI (34.95 vs. 47.24 ml/m^2^, + 35%, *p* = 0.008) were significantly higher than in no FACM cases.

**Fig. 2:**
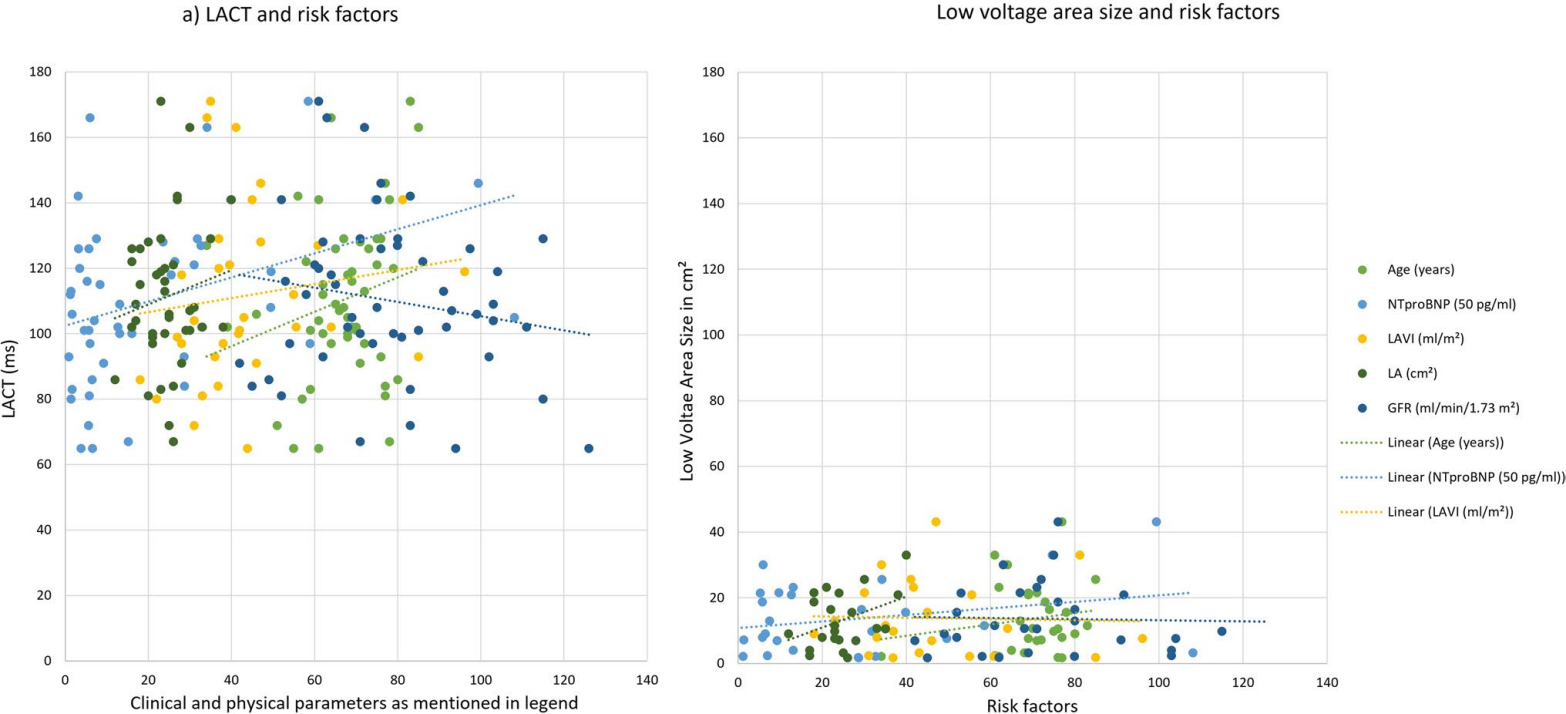
Correlation of clinical and physical parameters with LACT and LVA Size. In **a** LACT provides significant correlation with NTproBNP-levels (*p* = 0.40; *p* = 0.04) and LAVI (*p* = 0.28; *p* = 0.11) as well as moderate correlation to age (*p* = 0.20; *p* = 0.16). A strong cohesion (*p* = 0.40; *p* = 0.03) of low voltage area and LA (cm^2^) was detected in (**b**). *LAVI* left atrial volume index, *LA* left atrial planimetric area, *GFR* glomerular filtration rate, *NTproBNP* N-Terminal prohormone of brain natriuretic peptide

In estimating the fibrosis volume, the mean LVA size of FACM patients was calculated to an average of 13.65 ± 10.6 cm^2^. With this, regional differences appeared as the LVA was more extensive in the anterior (10.8 ± 7.0 cm^2^, *n* = 28) than in the posterior wall (6.50 ± 6.6 cm^2^, *n* = 9). The extent of the LVA correlated moderately with the left atrial planimetric area (*r* = 0.40, *p* = 0.033) and the CHA_**2**_DS_**2**_–VASc score (*r* = 0.29, *p* = 0.123). At the same time, there was no correlation found to LAVI (*r* = − 0.08, *p* = 0.678), NTproBNP levels (*r* = 0.14, *p* = 0.475), renal function (*r* = − 0.03, *p* = 0.583), bodyweight (*r* = − 0.16, *p* = 0.397), BMI (*r* = − 0.16, *p* = 0.400) and sex (*r* = 0.04, *p* = 0.815). The correlations between clinical risk factors and LVA are presented in Fig. 2. For internal validation, the cohesion of LVA and FACM was assessed. The data proved inherently consistent since the correlation was strong (*r* = 0.85, *p* < 0.001).

### Total left atrial conduction time

**Fig. 3:**
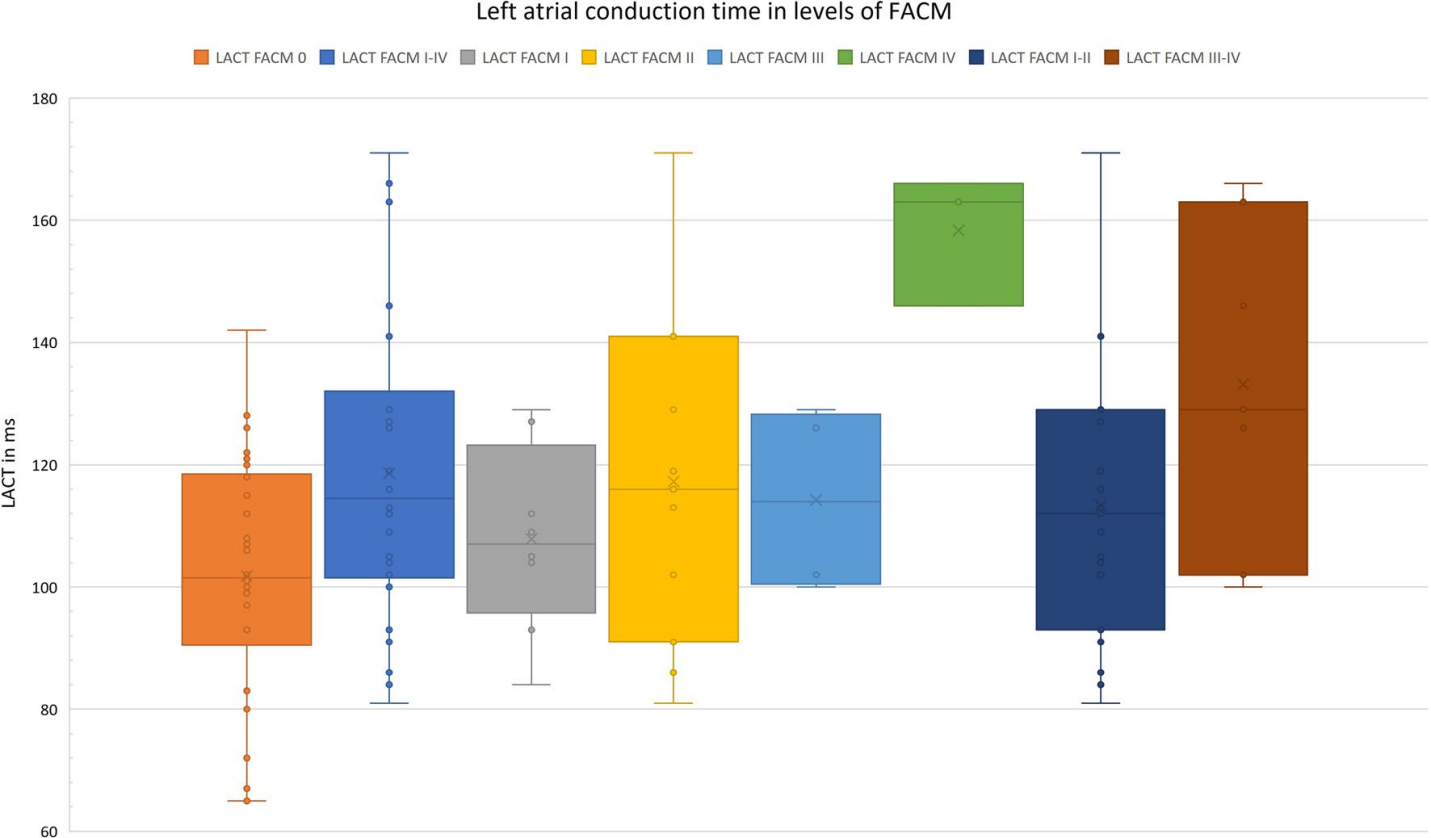
LACT of all patients categorized in FACM class. As a statistical significant trend, the LACT extends with higher grade of disease (*p* = 0.50). *LACT* left atrial conduction time, *FACM* fibrotic atrial cardiomyopathy

The overall LACT was calculated with an average length of 110.20 ± 24.42 ms (range 65 to 171 ms). For the different grades of FACM, the LACT differed significantly (F_(4,46)_ = 5.418, p = 0.001, *η*^2^ = 0.320, έ^2^ = 0.261). While no FACM patients presented a mean LACT of 101.40 ± 20.7 ms, the conduction duration was prolonged in FACM patients (I–IV) by nearly 17% to 118.65 ± 25.1 ms (*p* = 0.005). Bonferroni post-hoc analysis revealed a particularly pronounced difference between the LACT of no FACM and high-grade FACM cases (101.40 ± 20.7 vs. 133.1 ± 26.7 ms, + 31.2%, mean difference 31.74, *p* = 0.005, 95% CI [8.02, 55.46]) (cf. Fig. 3 and Table 2).

**Fig. 4:**
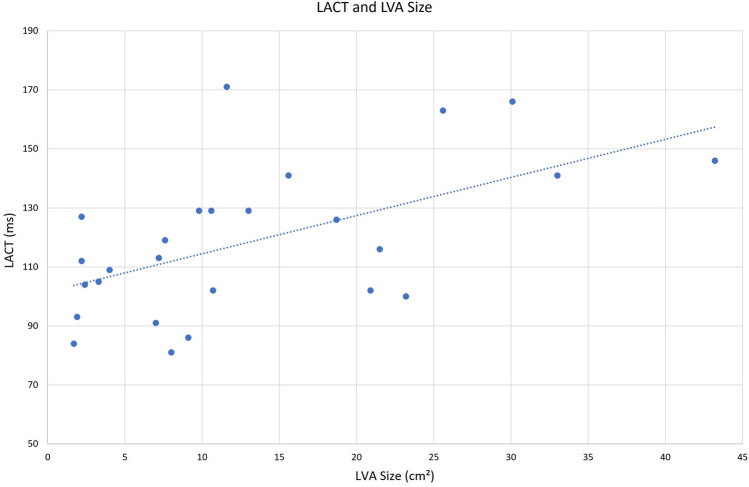
LACT and LVA Size plotted. The LACT elongates with LVA Size and thereby provides a highly significant correlation (*p* = 0.56). *LACT* left atrial conduction time, *LVA* low voltage area

Furthermore, LACT correlated significantly with the extension of LVA (*r* = 0.56, *p* = 0.002) (cf. Fig. 4). In our cohort, all physical dimensions corresponded negatively with the LACT. Body weight (*r* = − 0.46, *p* < 0.001) and body surface area (r = − 0.48, *p* < 0.001) met the criteria for relevant correlation, whereas BMI (*r* = − 0.19, *p* = 0.190) presented only little effect power with no statistical significance. In addition, data indicated relevant cohesion between LACT and NTproBNP levels (*r* = 0.40, *p* = 0.004) as well as CHA_**2**_DS_**2**_–VASc score (*r* = 0.33, p = 0.017). In contrast, significant associations of LACT with LAVI (*r* = 0.28, *p* = 0.107), left atrial planimetric area (*r* = 0.14, *p* = 0.360), and renal function (*r* =  −  0.17, *p* = 0.219) were rejected (cf. Fig. 2).

### Conduction velocities

**Fig. 5:**
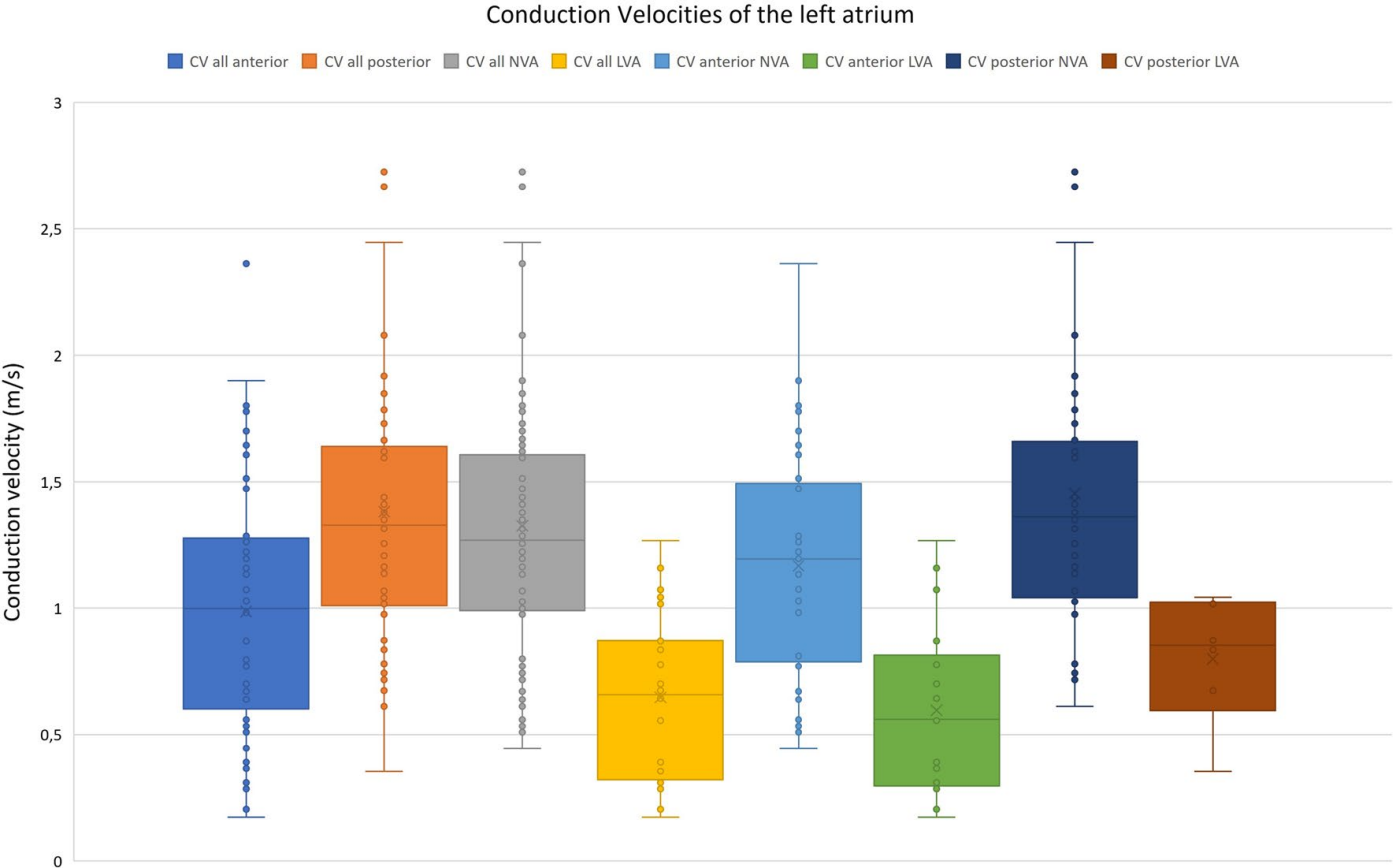
CV clustered by atrial wall side and voltage. CV are faster posterior than anterior (+ 40%, *p* = 0.000) and in NVA than in LVA (+ 105%, *p* = 0.000). This tendence applies to all subgroups. *CV* conduction velocities, *NVA* normal voltage area, *LVA* low voltage area

**Fig. 6:**
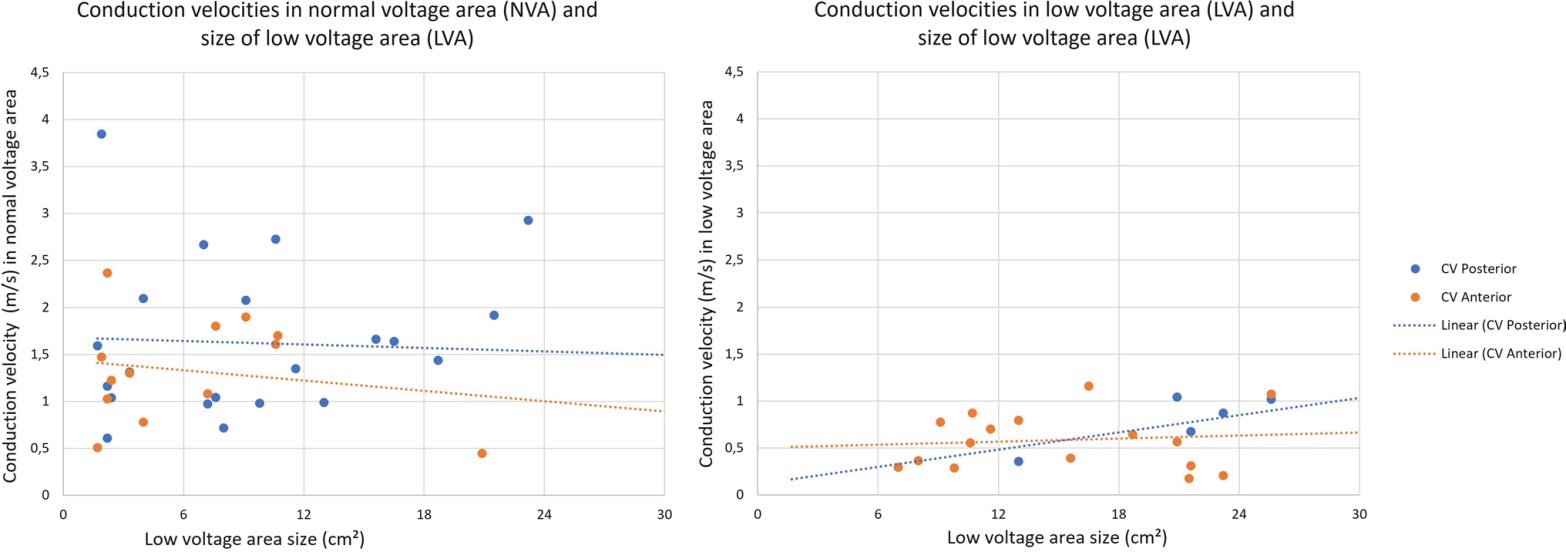
CV in NVA and LVA plotted to LVA. While the CV in NVA decreases, the CV in LVA increases with LVA size. This tendency is coherent in anterior and posterior subgroups although statistical significance was only proven by anterior CV in NVA (*r* =  − 0.36) and posterior CV in LVA (*r* = 0.68). It is important to notice, that the incline in last mentioned CV in LVA is defined by the single point at 13 cm^2^ and therefore to interpret conservatively. *NVA* normal voltage area, *LVA* low voltage area, *CV* conduction velocities, *LACT* left atrial conduction time

CV in anterior and posterior walls as well as in NVA and LVA are illustrated in Figs. 5 and 6. Exclusively considering voltage difference, conduction velocities in LVA were revealed to be 51% slower than in NVA (0.64 ± 0.33 vs. 1.33 ± 0.54 m/s, *p* < 0.001). Regarding only regional differences, we found CV in the anterior LA (0.98 ± 4.8 m/s, range 0.17–2.36 m/s) significantly slower than in the posterior LA (1.38 ± 0.6 m/s, range 0.36–3.85 m/s, − 29%, *p* < 0.001).

When considering regional and voltage characteristics, in NVA, the anterior conduction was 19.7% slower than the posterior (1.17 ± 0.44 vs. 1.45 ± 0.59 m/s, *p *= 0.007). A similar trend was recognized in low voltage areas since conduction in the anterior wall was 25.5% slower than in the posterior wall but failed to reach statistical significance (0.60 vs. 0.8 m/s, *p* = 0.096). However, in high-grade FACM, these regional conduction velocity differences between the anterior and posterior walls leveled out (0.75 vs. 0.80 m/s, *p* = 0.394). Furthermore, when comparing anterior wall conduction velocities in LVA, the Kruskal–Wallis test rejected relevant differences between low-grade and high-grade FACM patients (0.47 ± 0.24 vs. 0.75 ± 0.39 m/s, *p* = 0.110).

To examine whether FACM also impairs conduction properties in NVA, conduction velocities in the anterior and posterior walls of FACM patients were compared to those in no FACM patients by Mann–Whitney *U* tests. The distributions between no FACM and FACM did not differ in anterior (*p* = 0.471) and posterior (*p* = 0.674) walls. There was no significant conduction velocity difference in NVA between no FACM and FACM patients in the anterior (*U* = 174, *Z *= − 0.029, *p* = 0.977) and the posterior (*U* = 270, Z = − 0.361, *p* = 0.718) walls.

## Discussion

### Main findings

This data analysis provides evidence that Fibrotic Atrial Cardiomyopathy (FACM) significantly influences the left atrial conduction characteristics in patients with persistent AF. First, the total left atrial conduction time was prolonged with the FACM grade and the quantitative expanse of low voltage areas by up to 31% in high-grade disease. Second, compared to NVA, LVA showed a 51% conduction velocity reduction. Third, regional differences in conduction velocities were present in the left atrium. In normal voltage areas, anterior conduction seemed slower than posterior conduction. This variation was also seen in LVA but failed to reach statistical significance.

### Conduction properties in AF patients in comparison with other studies

Structural remodeling in patients with atrial fibrillation is linked to electrophysiological remodeling. As a result of atrial fibrogenesis, non-uniform anisotropic impulse propagation arises, and conduction heterogeneity increases. These factors might sustain AF by favoring reentry and AF driver anchoring [26, 27]. Slow conduction sites are prone to reentry formation and perpetuation of AF. Zheng et al. reported slower conduction velocities in AF patients (0.60 ± 0.12 m/s) compared to healthy controls (0.83 ± 0.13 m/s) and slower velocities in the LA compared to the right atrium (0.51 ± 0.11 m/s) [28]. In addition, despite the clinical evidence of AF, Sanders et al. have shown conduction slowing in patients with congestive heart failure compared to controls [29]. Structural and electrophysiologic changes in AF patients lead to worse outcomes after catheter ablation. AF recurrence after PVI was shown to be more likely to occur in patients with LVAs [3] as well as in patients with slower conduction velocities of the atrium [20].

Electrophysiological activation maps were previously used to estimate the conduction velocity and help localize slow conduction regions associated with cellular decoupling and fibrosis [30, 31]. Even without establishing an explicit voltage cutoff for “low voltage”, respectively fibrotic areas, other groups have additionally shown bipolar electrogram changes with fractionated and double potentials in low voltage areas (< 0.5 mV) [29, 32] and that rate-dependent CV slowing sites are predominantly confined to low-voltage zones (0.2–0.5 mV).

In line with our results, Miyamoto et al. showed a significant LACT prolongation in AF patients with the smallest electrogram amplitudes < 0.5 mV (which would refer to our FACM patients) to 107 ± 25 ms, while patients with the smallest electrogram amplitude of 0.75–1 mV were calculated with a faster LACT of 85 ± 13 ms [32]. In the present study, we have seen LACTs < 100 ms in only 27% of our maps. This finding might be explained by our strict selection of only persistent AF patients whereas in Miyamoto’s population only 20% of cases matched the criteria for persistent AF. Concerning local conduction velocities, Miyamoto et al. found significantly slower CV in LVAs compared with the non-LVA regions (0.8 ± 0.5 vs. 1.4 ± 0.6 m/s).

### Interpretation of conduction velocities

Since regional CVs depend on a multitude of variables, such as ion channel properties, cell-to-cell coupling, base rate, wavefront geometry, and muscle thickness, their interpretation is challenging [33]. Thus, several computer models of the atria have been developed [34–38]. However, data from high-resolution maps in a clinical procedure, especially in conjunction with evaluating LA substrate, are sparse. In contrast to the ventricular systems with their regular transmural fiber orientation, atrial morphology and its fiber alignment exhibit higher interindividual variability [39]. Therefore, we cannot exclude that deviations in muscle thickness and fiber orientation between the left atrial anterior and posterior walls affected our CV measurements. These confounders could potentially be responsible for the regional differences found in our analysis, even though we have taken the Coherent algorithm for wavefront direction into account.

### Ablation strategies for persistent AF

Individualized ablation concepts are needed to improve catheter ablation outcomes in persistent AF patients with an atrial substrate. A deeper understanding of atrial conduction characteristics as a functional aspect besides low voltage area detection might influence future ablation concepts in those patients.

Due to the moderate outcome results of persistent AF catheter ablation studies comparing substrate-based ablation approaches to PVI alone [9, 10, 40–42], some electrophysiologists have already dropped the idea of substrate mapping and more extended ablation concepts in those patients, particularly in first procedures. However, those studies did not prove lone PVI to be sufficient since efficacy is far from satisfying. These results highlight the need for improved individualized ablation strategies and stress that empiric ablation concepts (e.g., line sets) are not the optimal treatment for all persistent AF patients. Since low voltage areas are found in about 30–40% of persistent AF patients, only those should be classified as a target population for substrate-based ablation. Persistent AF patients without FACM will have similar results to paroxysmal AF patients with PVI alone.

Our results underline that the electrophysiological conduction characteristics change significantly in areas with the commonly used voltage cutoffs and mark them as more pro-arrhythmogenic. This emphasizes that low-voltage regions still need to be targeted. Thus, to evaluate their efficacy, randomized trials of individual substrate-based ablation protocols considering the structural conduction differences presented in this report are required.

The recently published ERASE-AF trial was the first prospective, randomized, multicenter trial that indicated improved arrhythmia-free survival by utilizing a voltage area-based ablation approach with a 38% risk reduction for atrial arrhythmia recurrence in 324 patients with persistent AF [19]. More such investigations are needed. In addition, transmural lesions are an important goal that is hard to achieve using current technologies without increasing complication rates (e.g., atrioesophageal fistulas while ablating the posterior wall). New technologies such as non-thermal ablation (pulsed-field ablation) going beyond PVI might help to treat the target regions effectively.

Preprocedural screening would be desirable to detect low voltage areas or left atrial conduction impairment to allocate patients to the most successful mapping/ablation strategy. Unfortunately, for imaging-based substrate/fibrosis detection, we still need to overcome the technical limitations of atrial magnetic resonance imaging. In comparison, screening for long atrial conduction times with noninvasive mapping tools (e.g., with multi-electrode vests) might be feasible in the future and might be added or combined with AI-based algorithms using different standard clinical parameters (ECG, echo measurements, biomarkers, etc.) to improve decision making for patients and planning catheter ablation procedures.

## Limitations

The investigation’s main limitations are its retrospective study design and the small patient population, especially in high-grade diseases. The latter is due to the narrow selection criteria since only high-quality sinus rhythm maps (> 1000 points) of patients with no prior substrate modification yet clinical evidence of persistent AF were eligible for enrollment. In addition, only one mapping system was allowed to avoid confounders. Moreover, the use of conventional mapping catheters (circular, ten poles) may have influenced the distribution and size of LVAs [43]. Another limitation is that voltage cutoffs for LVAs are still under investigation, and histological evidence for the definition of LVAs/FACM is lacking. Further studies are desirable here. Our voltage cutoff < 0.5 mV is conventionally regarded as LVA by other groups, while it is known that areas with voltage > 0.5 mV could still be abnormal. Besides that, we did not assess electrogram properties (e.g., fractionation) or perform rate-dependent measurements. Statistical regression models were not applicable due to the small sample size.

## Conclusion

FACM has a significant influence on LA conduction characteristics in AF patients. LACT was significantly prolonged in AF patients with an atrial substrate, showed an increase of 31% in high-grade FACM, and correlated positively with the extent of LVA size. Moreover, compared to areas with a normal bipolar voltage, LVA presented a 51% CV reduction. Furthermore, regional CVs differed between the LA anterior and posterior walls. Those findings might impact the development of individualized substrate-based ablation strategies in catheter ablation of FACM patients.
